# Feasibility, safety and effectiveness of robot-assisted retroperitoneal partial adrenalectomy with a new robotic surgical system: A prospective clinical study

**DOI:** 10.3389/fsurg.2023.1071321

**Published:** 2023-02-22

**Authors:** Jie Dong, Ruoyu Ji, Guanghua Liu, Jingmin Zhou, Huizhen Wang, Weifeng Xu, Zhigang Ji, Liang Cui

**Affiliations:** ^1^ Department of Urology, Peking Union Medical College Hospital, Peking Union Medical College, Chinese Academy of Medical Sciences, Beijing, China; ^2^ Department of Operation Room, Peking Union Medical College Hospital, Peking Union Medical College, Chinese Academy of Medical Sciences, Beijing, China; ^3^ Department of Urology, Civil Aviation General Hospital, Civil Aviation Medical College of Peking University, Beijing, China

**Keywords:** partial adrenalectomy, robotic surgery, complication, benign adrenal tumor, retroperitoneal

## Abstract

**Objectives:**

To evaluate the feasibility, safety and efficacy of the newly developed KD-SR-01® robotic system for retroperitoneal partial adrenalectomy.

**Subjects and Methods:**

We prospectively enrolled patients with benign adrenal mass undergoing KD-SR-01® robot-assisted partial adrenalectomy in our institution from November 2020 to May 2022. Surgeries were performed *via* a retroperitoneal approach using the KD-SR-01® robotic system. The baseline, perioperative and short-term follow-up data were prospectively collected. A descriptive statistical analysis was performed.

**Results:**

A total of 23 patients were enrolled, including nine (39.1%) patients with hormone-active tumors. All patients received partial adrenalectomy *via* the retroperitoneal approach without conversions to other procedures. The median operative time was 86.5 min [interquartile range (IQR), 60.0–112.5] and the median estimated blood loss was 50 ml (range, 20–400). Three (13.0%) patients developed Clavien-Dindo grade I-II postoperative complications. The median postoperative stay was 4.0 days (IQR, 3.0–5.0). All surgical margins were negative. The short-term follow-up demonstrated complete or partial clinical and biochemical success as well as absence of imaging recurrence in all patients with hormone-active tumors.

**Conclusions:**

Initial results illustrate that the KD-SR-01® robotic system is safe, feasible and effective for the surgical management of benign adrenal tumors.

## Introduction

Total adrenalectomy is recommended as the first-line management for benign adrenal mass requiring surgical resection (1). With the development of minimally invasive technique, minimally invasive partial adrenalectomy (PA) is expected to be an alternative organ-sparing procedure with the aim of preserving adrenal function, especially for patients with bilateral tumors. Although feasible and safe, evidence regarding the reliability and benefit of PA is still in exploration, though a systematic review suggested that PA was correlated with infrequent recurrence and high steroid independence (2). Endorsement of robotic technologies may extend indications of PA, in analogy with partial nephrectomy in renal tumors (3). Recent studies illustrated that robot-assisted partial adrenalectomy (RAPA) promises encouraging perioperative and functional outcomes in small benign and hormonal active adrenal tumors (4–6). However, the most frequently used DaVinci® robot (Intuitive Surgical, Sunnyvale, CA, USA) for RAPA was challenged by its longer operative time and remarkably higher costs especially for patients in developing countries (7, 8). With an increasing application of RAPA for benign adrenal tumors in China, a self-developed robotic system is needed to address this issue. Recently, a domestic robotic platform called KD-SR-01® (SuZhou Kang Duo Robot Co., Ltd., Suzhou, China) has been developed. The feasibility, safety and efficacy of KD-SR-01® has been confirmed in radical prostatectomy and partial nephrectomy, which additionally provided short docking time and ergonomic benefits (9, 10). Nevertheless, its performance in RAPA has not been assessed. Therefore, our study aimed to evaluate the feasibility, safety and efficacy of KD-SR-01® for retroperitoneal RAPA.

## Materials and methods

### Patients

The study was conducted in accordance with the Declaration of Helsinki. The study was approved by the Ethics Review Committee of Peking Union Medical College Hospital (PUMCH) with a registration number of HS-2410. Informed consent was obtained from all patients. Included in this prospective study were patients aged between 18 and 75 years, who underwent RAPA for adrenal mass in PUMCH from November 2020 to May 2022. Indications for RAPA were clinically benign adrenal tumors with a long diameter of <5 cm on imaging. Exclusion criteria included: (1) pregnancy; (2) severe systemic diseases; (3) combined with other urological diseases which may interfere the operation such as tumors, malformations and tuberculosis; (4) malignant or recurrent tumors.

### Preoperative management

All patients underwent enhanced abdomen computed tomography (CT) or magnetic resonance imaging (MRI) to assess location and size of the mass. Laboratory tests including complete blood count, liver function test, renal function test, coagulation test, electrolyte test as well as a full set of endocrine tests were performed before surgery. Perioperative managements of hormone-active tumors were performed according to guidelines (11–13).

### Robotic platform

The KD-SR-01® robotic system passed the special channel of National Medical Products Administration (NMPA) for innovative medical devices and was allowed to enter the clinical trial in 2019. KD-SR-01® has been approved for registration by NMPA of in June 2022.

**Figure 1:**
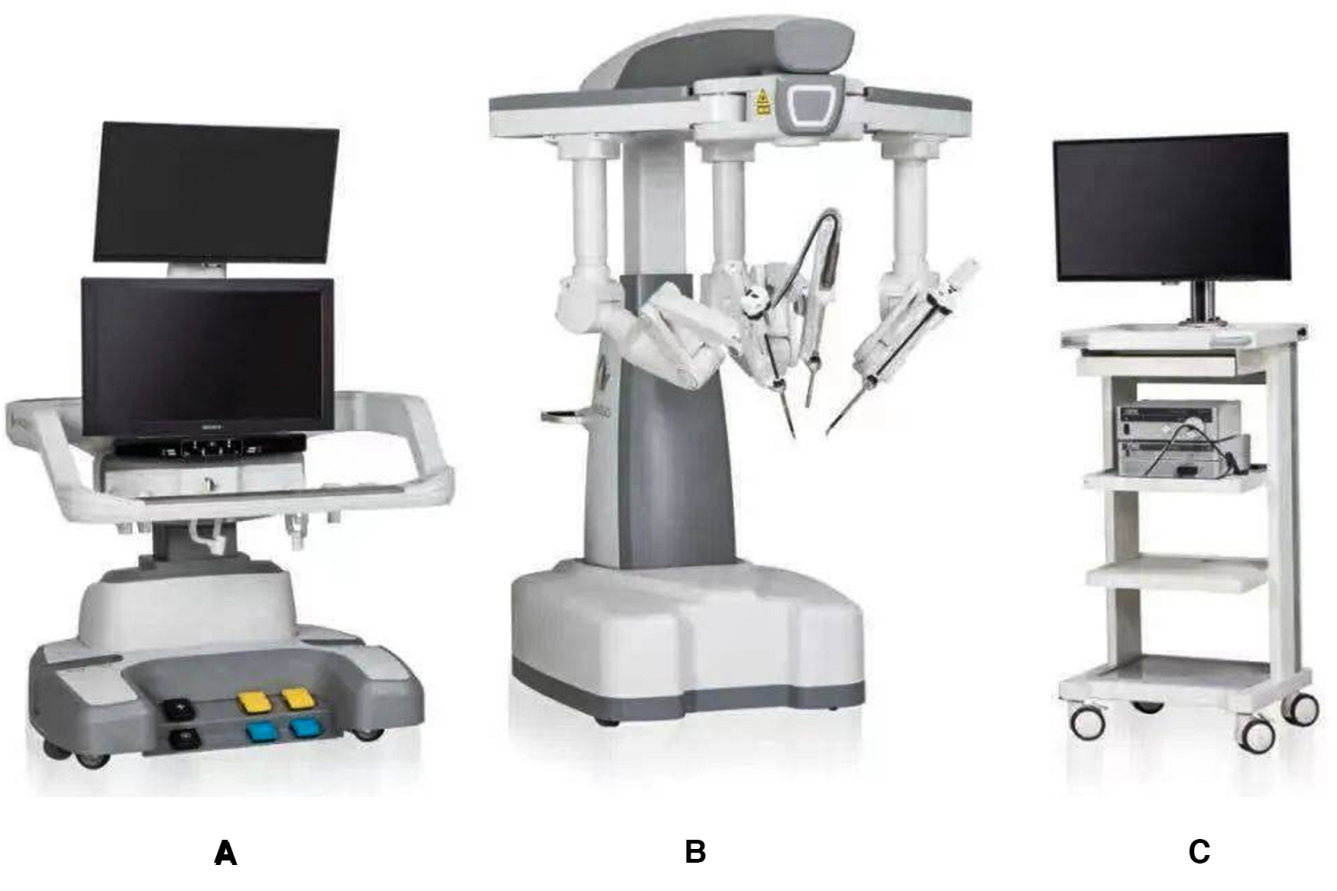
The KD-SR-01® robotic system which composes of (**A**) a surgical console, (**B**) a patient cart and (**C**) a 3D imaging system.

The KD-SR-01® robotic system is composed of a surgical console, a patient cart and a 3D imaging system (Figures 1A–1C). The surgical console is integrated with two master manipulators and a 3D high-definition monitor, combined with passive polarizing glasses, enabling surgeons to precisely and synchronously control the surgical arms and instruments without flexion of the neck (10) The patient cart is designed as a three-arm system, which has seven degrees of freedom for movements and can filter out tremors of the hands. The imaging system adopts a modular interface, which can match various 3D laparoscopic display systems. In this study, Karl Storz IMAGE1 SD3-LinkTM laparoscopic systems were used with 30° 10-mm three-dimensional video laparoscopes.

### Surgical team and technique

The surgical team contained two expert surgeons, who have altogether performed more than 300 cases of RAPA using the DaVinci® system. Prior to the initiation of the study, two surgeons and two operating room nurses were exclusively trained for three days by performing surgical procedures on porcine models utilizing the KD-SR-01® robotic system.

**Figure 2:**
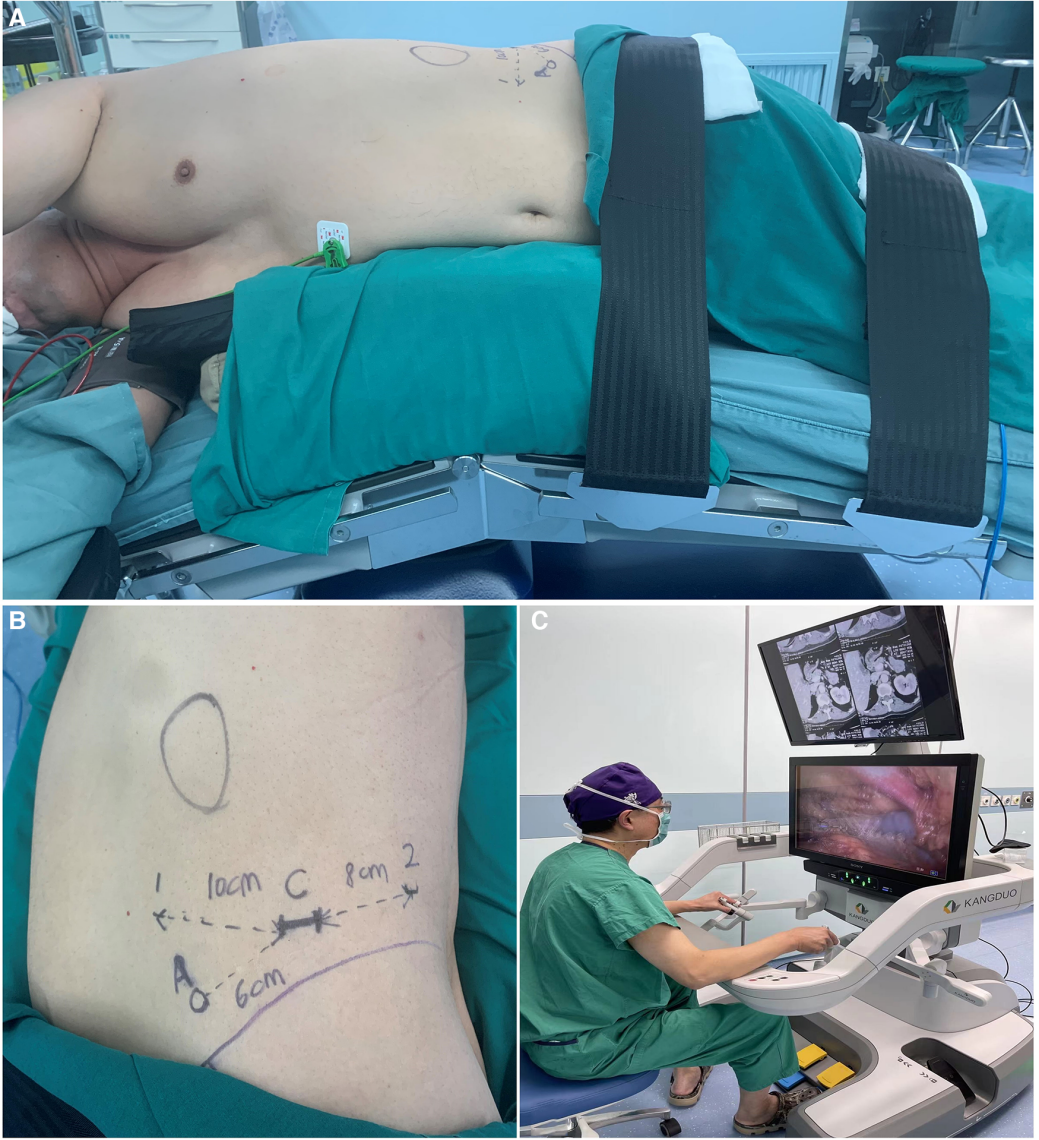
Patient position, ports placement and view of console in robot-assisted partial adrenalectomy using the KD-SR-01® robotic system. (**A**) the patient was placed in a lateral flank position. (**B**) the camera port (**C**) was placed 2 cm above the iliac crest (purple line) on the midaxillary line. Two ports for robotic instruments were placed 4–5 cm above the iliac crest on the anterior axillary line (1) and 2–3 cm above the iliac crest on the posterior axillary line (2), respectively. The distance between the camera port (**C**) and two ports for robotic arms were 8–10 cm, avoiding chance collisions. The assistant port (**A**) was placed 3–4 cm below the midpoint of the camera port (**C**) and the anterior operating port (1). (**C**) an open console enables surgeons to operate without flexion of necks.

**Figure 3:**
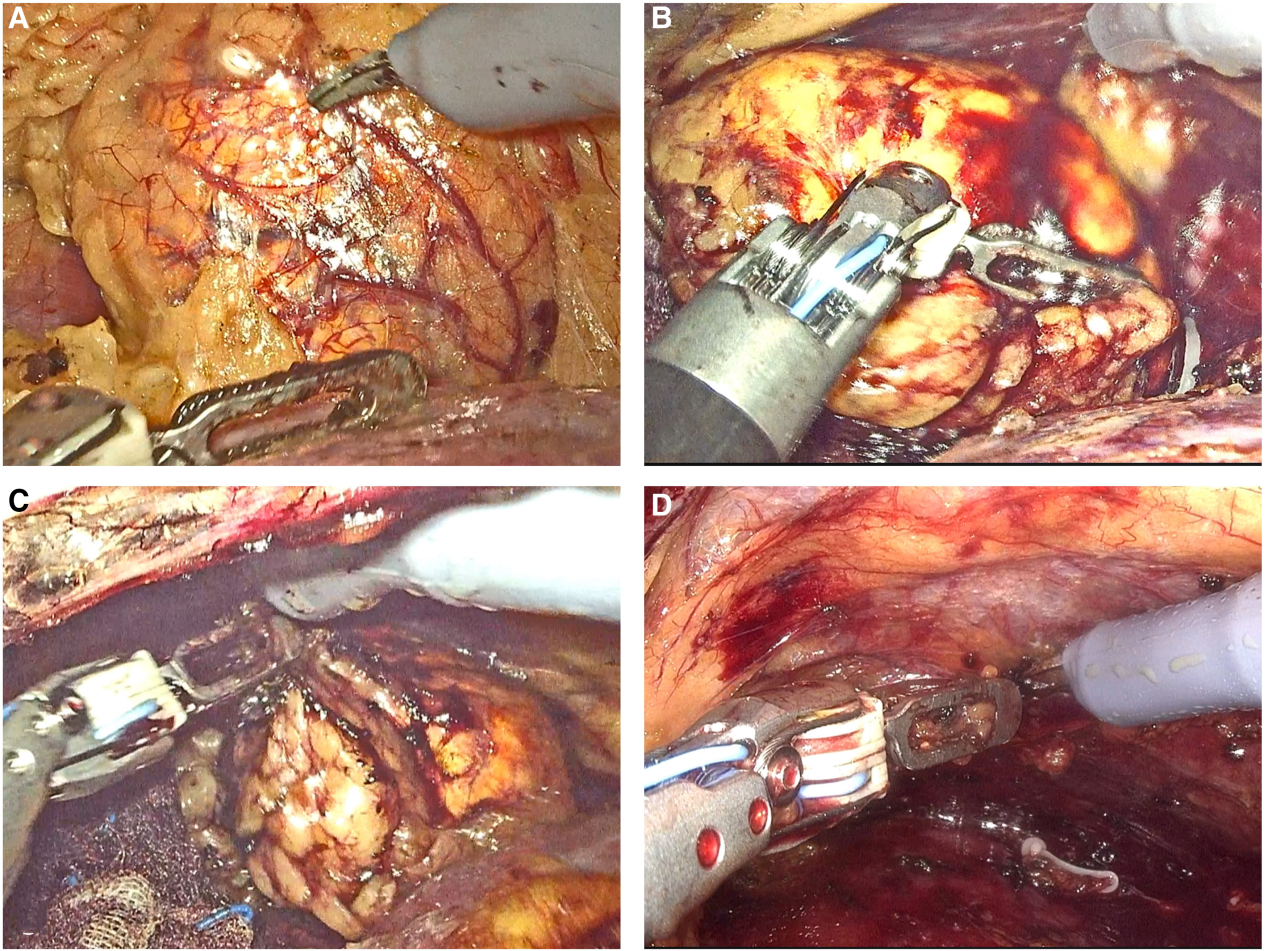
Surgical techniques of robot-assisted partial adrenalectomy using the KD-SR-01® robotic system. (**A**) exposing the adrenal mass. (**B**) mobilizing the adrenal mass from the dorsal site. (**C**) enucleated the mass with an integrated tumor margin. (**D**) confirming complete hemostasis.

All surgeries were performed *via* a retroperitoneal approach. After general anesthesia induction, the patient was placed in a lateral flank position (Figure 2A). A 2-cm incision was made at the midaxillary line 2 cm above the iliac crest, through which the retroperitoneal cavity was created by balloon dilation. After a pneumoperitoneum of 14 mmHg was established, a 30° 3D video laparoscope was introduced through the first incision. Then, two 10-mm robotic cannulas used as monopolar curved scissor and bipolar curved forceps were placed under direct vision at the anterior and posterior axillary lines above the iliac crest, respectively. Another assistant 10-mm trocar was placed between the camera port and the anterior port (Figure 2B). Robotic arms were then docked and were advanced into the extraperitoneal space under direct vision by an assistant surgeon. The operation was then conducted by the lead surgeon at the open console (Figure 2C). After dissociation of the kidney, the ventral side of the adrenal gland was then dissected between the superior renal pole fat sac and the Gerota fascia with the aim of exposing the adrenal mass (Figure 3A). The mass was then progressively mobilized at the level of the upper pole of the kidney and from the dorsal side, respectively (Figure 3B). After the mass is sufficiently exposed, it is completely enucleated from surrounding adrenal tissues with an integrated tumor margin (Figure 3C). For masses adjacent to the central vein of adrenal gland, the adrenal vein was ligated and excised. The surgical bed was checked for hemostasis and the remnant adrenal margins were sutured (Figure 3D) before the removal of the excised tumor with a specimen bag.

### Data collection and assessment

Study data were collected from the electronic medical record system of PUMCH. Collected perioperative data were demographic variables including age, sex, body mass index (BMI) and American Society of Anesthesiologists (ASA) score (14); baseline physical examination results including systolic blood pressure (SBP), diastolic blood pressure (DBP) and body temperature; baseline laboratory results including complete blood count, liver and renal function tests, electrolyte test as well as a full set of endocrine tests.

Collected intraoperative data were preparation time, docking time, console time, total operative time (OT), estimated blood loss (EBL) and intraoperative complications. The docking time was defined as the interval from the movement of the robotic cart to docking of the last canula to the corresponding arm. The console time was defined as the time from robot docking to robot undocking.

Collected postoperative data were pathology findings including tumor size, histology and margin status, postoperative physical examination results, postoperative laboratory results, admission to intensive care unit (ICU) and ventilator use, postoperative complications, time to first flatus and defecation, time to removal of urinary catheter and drainage tube, use of analgesics and antibiotics, length of stay (LOS) and 30-day readmission.

The estimated glomerular filtration rate (eGFR) was calculated by the CKD-EPI formula (15). Complications were graded using the Clavien-Dindo classification system and grade III-IV complications were defined as major complications (16). Diagnosis and outcome measures of hormone-active tumors were assessed according to relevant consensus and guidelines (11, 13, 17, 18).

The ergonomics were assessed by using the Subjective Mental Effort Questionnaire (SMEQ) and the Local Experienced Discomfort (LED) scale after each surgery by the surgical team (19). The SMEQ score ranges from 0 to 150 and lower scores indicated less mental effort. The LED scale evaluates user comfort with a score of 0 to 10 points and lower scores indicated less discomfort.

### Statistical analyses

Continuous variables were described as median [interquartile range (IQR), p25–p75] or median (range). Categorical variables were described as absolute value and percentages. Continuous variables were compared by Mann-Whitney U test. Analyses were performed using SPSS 25.0 for windows (SPSS institute, Chicago, IL, USA) and *P* value < 0.05 was considered statistically significant.

## Results

### Patient characteristics

Altogether, 23 patients, including 10 male and 13 female patients met the inclusion and exclusion criteria and were thus enrolled, whose baseline characteristics were summarized in Table 1. The median age, BMI and preoperative eGFR were 49.0 years (range, 22–67), 23.8 kg/m^2^ [interquartile range (IQR), 20.8–27.0] and 94.0 ml/min/1.73 m^2^ (IQR, 82.4–105.7), respectively. Six (26.1%) patients had a history of abdominal surgery. Nine (39.1%) patients had hormone-active tumor, including five (21.7%) aldosterone-producing adenoma (APA), two (8.7%) cortisol-producing adenoma (CPA) and two (8.7%) pheochromocytoma (PHEO). The median long diameter of tumors was 2.8 cm (IQR, 1.5–3.5).

**Table 1:** Baseline characteristics of patients undergoing robot-assisted partial adrenalectomy.

Patients (*n*)	23
Median age, year (range)	49.0 (22–67)
Male/female	10/13
Median BMI, Kg/m^2^ (IQR)	23.8 (20.8–27.0)
Median ASA score (Range)	2 (1–3)
Abdominal surgery history (*n*, %)	6 (26.1)
Median preoperative eGFR, ml/min/1.73 m^2^ (IQR)	94.0 (82.4–105.7)
Hormone-active tumors (*n*, %)	9 (39.1%)
Median long diameter of tumor, cm (IQR)	2.8 (1.5–3.5)

BMI, body mass index; IQR, interquartile range; ASA, American Society of Anesthesiologists; eGFR, estimated glomerular filtration rate.

### Perioperative and pathology outcomes

All patients received partial adrenalectomy *via* the retroperitoneal approach. The median OT was 86.5 min (IQR, 60–112.5), of which the median preparation time, docking time and console time were 15.3 (IQR, 10.0–17.3), 4.3 (IQR, 3.4–5.6), 46.5 (IQR, 35.1–59.0) minutes, respectively (Table 2). The median EBL was 50 ml (range, 20–400). No case was converted to open or received intraoperative transfusion. Two (8.7%) patients with PHEO were routinely transferred to ICU for one day.

**Table 2:** Perioperative and pathological outcomes of patients undergoing robot-assisted partial adrenalectomy.

Patients (*n*)	23
**Intraoperative outcomes**	
Side (left/right)	13/10
Median operative time, min (IQR)	86.5 (60.0–112.5)
Median preparation time, min (IQR)	15.3 (10.0–17.3)
Median docking time, min (IQR)	4.3 (3.4–5.6)
Median console time, min (IQR)	46.5 (35.1–59.0)
Median EBL, ml (range)	50 (20–400)
Conversion to open (*n*, %)	0 (0.0%)
Transfusion (*n*, %)	0 (0.0%)
ICU transfer (*n*, %)	2 (8.7%)
**Postoperative outcomes**	
Complications (*n*, %)	3 (13.0%)
Claviene-Dindo grade I	2 (8.7%)
Claviene-Dindo grade II	1 (8.7%)
Fever (*n*, %)	9 (39.1%)
Absorption fever (*n*, %)	8 (34.8%)
Antibiotic use (*n*, %)	1 (4.3%)
Incisional pain (*n*, %)	17 (73.9%)
Oral tramadol hydrochloride for pain relief (*n*, %)	2 (8.7%)
Median time to first flatus, day (range)	2 (1–3)
Median time to first stool dropping, day (range)	3 (2–4)
Median time of urinary catheter placement, day (range)	1 (1–3)
Median time of perirenal drainage, day (range)	3 (2–4)
Median perirenal drainage volume, ml (IQR)	90.0 (65.0–131.0)
In POD 1, ml (IQR)	50.0 (30.0–95.0)
In POD 2, ml (IQR)	25.0 (15.0–50.0)
Median decrease in hemoglobin, g/L (IQR)	10.0 (2.0–20.0)
Median LOS, day (IQR)	10.0 (8.0–12.0)
Median postoperative LOS, day (IQR)	4.0 (3.0–5.0)
Readmission in 30 days, *n* (%)	0 (0.0%)
**Pathological outcomes**	
Negative margin, *n* (%)	23 (100.0%)
Benign lesion, *n* (%)	23 (100.0%)
Adenoma, *n* (%)	19 (82.6%)
Pheochromocytoma, *n* (%)	2 (8.7%)
Adrenocortical nodular hyperplasia, *n* (%)	2 (8.7%)

IQR, interquartile range; EBL, estimated blood loss; ICU, intensive care unit; POD, postoperative day; LOS, length of stay.

Altogether, three (13.0%) patients developed postoperative complications, including one (4.3%) grade II complication of one patient requiring antibiotic treatment due to postoperative fever. 17 (73.9%) patients underwent incisional pain, and two (8.7%) of them received weak opioids for analgesia. During hospitalization, the median time to first flatus and stool dropping were 2 days (range, 1–3) and 3 days (range, 2–4). The median time of urinary catheter placement and perirenal drainage was 1 day (range, 1–3) and 3 days (range, 2–4). The overall perirenal drainage volume was 90.0 ml (IQR, 65.0–131.0), which declined from 50.0 ml (IQR, 30.0–95.0) on postoperative day 1 to 25.0 ml (IQR, 15.0–50.0) on postoperative day 2. The median LOS and postoperative LOS were 10.0 days (IQR, 8.0–12.0) and 4.0 days (IQR, 3.0–5.0), respectively. No patient was readmitted within 30 days.

According to the pathology findings, all surgical margins are negative. The removed masses, including 19 (82.6%) adenoma, two (8.7%) pheochromocytoma and two (8.7%) adrenocortical nodular hyperplasia pathologically.

### Follow-up for hormone-active patients

The short-term postoperative follow-up for hormone-active patients was performed at a median of 3.3 months (2.4–3.9) after discharge (Table 3). The follow-up was available in seven (77.8%) patients and two were unable to come for their visit due to personal reasons.

**Table 3:** Preoperative and follow-up data of patients with hormone-active tumors.

	APA 1	APA 2	APA 3	APA 4	APA 5	CPA 1	CPA 2	PHEO 1	PHEO 2
Age/Gender	49/F	43/M	67/F	47/F	63/M	22/F	32/F	52/F	57/M
**Preoperative data without medication**									
SBP/DBP max (mmHg)	180/100	200/130	180/110	170/100	180/100	130/85	145/100	170/105	160/100
Serum potassium (mmol/; n.v. 3.5–5.5)	2.7	2.6	1.8	2.4	3.0	3.8	3.9	4.2	3.6
Aldosterone level (standing, ng/ml; n.v. 6.5–29.6)	17.14	18.52	27.04	21.20	21.32	10.96	-	14.54	10.25
PRA (standing, ng/ml h; n.v. 0.93–6.56)	0.01	0.03	0.01	0.06	0.01	0.63	-	0.48	1.62
ARR (n.v. < 30)	1714	617	2704	353	2132	17	-	30	6.33
Cortisol (8 am, μg/ml; n.v. 4–22.3)	17.7	-	19.5	17.8	21.3	20.7	26.1	9.2	5
ACTH (8 am, pg/ml; n.v. 0–46)	<5	21.9	20.6	35.5	30.8	<5	<1	12.8	1.1
Urinary cortisol (*μ*g/24 h; n.v. 12.3-103.5)	168.8	153.1	46.9	-	82.4	374.4	641.5	55.1	49.7
Urinary NE (μg/24 h; n.v. 16.7–40.9)	-	-	-	16.1	-	-	-	326.5	42.5
NM (nmol/L; n.v. < 0.3)	0.04	0.13	-	-	0.08	0.11	-	0.75	0.21
NMN (nmol/L; n.v. < 0.9)	0.31	0.32	-	-	0.23	0.11	-	3.84	14.42
**Follow-up data**									
SBP/DBP max (mmHg)	135/75	140/90	135/85	130/80	145/90	120/70	135/85	120/80	120/70
Serum potassium (mmol/; n.v. 3.5–5.5)	4.2	4.0	3.5	3.6	4.5	3.8	3.8	3.7	3.4
Aldosterone level (standing, ng/ml; n.v. 6.5–29.6)	13.08	14.79	-	-	13.86	-	-	-	-
PRA (standing, ng/ml h; n.v. 0.93–6.56)	0.22	0.51	-	-	0.05	-	-	-	-
ARR (n.v. < 30)	59	29	-	-	277	-	-	-	-
Cortisol (8 am, μg/ml; n.v. 4–22.3)	-	-	-	-	-	14.6	21.9	17.1	-
ACTH (8 am, pg/ml; n.v. 0–46)	-	-	-	-	-	6.2	4.4	32.4	-
Urinary cortisol (μg/24 h; n.v. 12.3–103.5)	-	-	-	-	-	81.9	152.7	-	-
Urinary NE (μg/24 h; n.v. 16.7–40.9)	-	-	-	-	-	-	-	19.1	13.8
NM (nmol/L; n.v. < 0.3)	-	-	-	-	-	-	-	0.08	0.11
NMN (nmol/L; n.v. < 0.9)	-	-	-	-	-	-	-	0.37	0.48
Antihypertensive medications	No	Yes	Yes	Yes	Yes	No	No	No	Yes
Potassium supplementation	No	No	No	No	Yes	No	No	No	No
Cortisol supplementation	No	No	No	No	No	Yes	Yes	No	No
**Outcome measures**									
Clinical success	Complete	Partial	Partial	Partial	Partial	Complete	Complete	Complete	Partial
Biochemical success	Partial	Complete	-	-	Partial	Complete	Partial	Complete	Complete
Imaging recurrence	No	No	-	-	No	No	No	No	No

APA, aldosterone-producing adenoma, CPA, cortisol-producing adenoma; PHEO, pheochromocytoma; SBP, systolic blood pressure; DBP, diastolic blood pressure; n.v., normal value of our institution; PRA, plasmatic renin activity; ARR, aldosterone-renin ratio; ACTH, adreno-cortico-tropic-hormone; NE, norepinephrine; MN, metanephrine; NMN, normetanephrine.

In the five APA patients, the postoperative SBP (175 mmHg (IQR, 180–190) vs. 135 mmHg (IQR, 133–143), *P* = 0.042) and DBP (100 mmHg (IQR, 100–120) vs. 85 mmHg (IQR, 78–90), *P* = 0.042) decreased significantly compared to baseline. One patient stopped taking antihypertensive drug, and the remaining four patients underwent reductions in number or dose of antihypertensive drugs to varying degrees. Serum potassium elevated from 2.6 mmol/L (IQR, 2.1–2.9) to 4.0 mmol/L (IQR, 3.6–4.4) (*P* = 0.042). One patient still received potassium supplementation discontinuously. For the three APA patients who came for follow-up, the aldosterone-renin ratio (ARR) declined from 1,714 to 59, 617 to 29 and 2,132 to 277, respectively.

In the two CPA patients, the postoperative blood pressure decreased from 130/85 to 120/70 mmHg and 145/85 to 135/70 mmHg, respectively. The cortisol (8 am) level decreased from 20.7 to 14.6 *μ*g/ml and 26.1 to 21.9 *μ*g/ml, respectively. The urinary cortisol level decreased from 374.4 to 81.9 *μ*g/24 h and 641.5 to 152.7 *μ*g/24 h, respectively. The adreno-cortico-tropic-hormone (ACTH) level increased accordingly.

In the two PHEO patients, the postoperative blood pressure (SBP/DBP) decreased from 170/105 to 120/80 mmHg and 160/100 to 120/70 mmHg, respectively. One patient still takes antihypertensive drug. Relevant endocrine measures declined to the normal range.

### Ergonomics and user comfort

The median SMEQ score (scoring from 0 to 150 points) was 45 points (range, 20–90), indicating some efforts. The median LED score (scoring from 0 to 10 points) was 1.7 (range, 0–7), indicating some discomforts.

## Discussion

Currently, partial adrenalectomy is increasingly being applied in the surgical management for benign adrenal mass. With rapid advances in robotic technology, RAPA has been proposed as an approach that enables patients to benefit more from minimally invasive surgery. The safety, feasibility and effectiveness of DaVinci® robot, the most frequently used surgical robot worldwide, in RAPA have been demonstrated by previous studies (5, 6). However, its prohibitive expense limited adoptions of RAPA in China. Therefore, a self-developed robotic system is of high clinical need to address this issue. In this study, we prospectively evaluated the feasibility, safety and efficacy of KD-SR-01® robotic system for RAPA based on 23 patients. Our preliminary results demonstrate that RAPA could be performed safely and effectively based on the domestic KD-SR-01® robot.

Utilizing the KD-SR-01® robotic system, all surgeries were performed successfully without conversion to open and intraoperative transfusion. The intraoperative EBL was also well controlled. The median and mean OT in our study was 86.5 and 94.3 min respectively, which was comparable with and even shorter than that recently reported by DaVinci® robot -assisted adrenalectomy (ranged from 98 to 128 min) (7, 20). A previous meta-analysis based on 27 studies reported a significantly longer OT in robotic adrenalectomy compared with laparoscopic adrenalectomy, which was assigned to the relatively long docking and undocking time (21). Here, the median docking time of KD-SR-01® was 4.3 min, thus largely saving the time for docking. Another contributing factor to the shorter OT in this study could be extensive experience of our surgical team in DaVinci® robot-assisted partial adrenalectomy, suggesting that there is little difficulty in transitioning from other robotic systems to KD-SR-01® system. The newly-develpoed robotic system also achieved good results in assessments of ergonomics and user comfort. Some minor problems such as the absence of naked eye 3-dimensional technology and tactile feedback system still exist and remains to be improved. All in all, the above-mentioned results all illustrated the feasibility of the self-developed robotic system.

The minimally invasive nature of RAPA is considered to correlate with fewer perioperative complications. In the present study, the overall complication rate was 13.0%, slightly higher than that reported in robot-assisted total adrenalectomy (7, 21, 22). However, RAPA is more complex in surgical techniques. In addition, some studies only reported Clavien–Dindo grade II-IV postoperative complications and our rate might be biased by the small sample size (7, 22), thus the complication rates could not be directly compared. Patients developing Clavien–Dindo grade I-II complications recover rapidly after symptomatic treatments. There was no major (Clavien-Dindo graded ≥ III) perioperative complication in our patients. These data, taken together, underline the safety of KD-SR-01® robotic system for RAPA. In addition, the newly developed system enabled a rapid recovery, illustrated by the early recovery of gastrointestinal tract and the shorter period of urinary catheter placement and perirenal drainage.

Regarding the short-term efficacy, all surgical margins are negative, and no 30-day readmission occurred in the present study. Imaging recurrence was also absent. The short-term follow-up demonstrated complete or partial clinical and biochemical success in all patients (13, 17, 18). However, the present efficacy assessment was only qualitatively conducted based on consensus and guidelines. A precise definition of clinical success was complicated as involvements of primary hypertension could not be completely excluded (17). Furthermore, long-term efficacy was not assessed. longer To address this issue, introducing a well-designed scoring system (23) based on a longer follow-up could help to evaluate the long-term efficacy quantitatively and precisely.

To the best of our knowledge, our study is the first to evaluate the feasibility, safety and efficacy of KD-SR-01® for RAPA. The prospective nature strict follow-up improves the reliability of our results. The KD-SR-01® surgical robot is completely self-developed and has all independent intellectual property rights. Although the price has not yet been officially determined, it is estimated that the price was about 25% to 30% of that of the DaVinci® robot based on the experience of our center, largely reducing the economic burden of RAPA for Chinese patients. Nevertheless, the current study has some shortcomings. First, the small sample size, a wide variability of patient ages, complete Chinese population and single-center data limit generalizations and interpretations of our findings. Second, the short follow-up period does not allow analyses for long-term efficacy. Third, the pricing has not been completed which precluded the cost analysis. Fourth, the single-arm design limits the comparison of KD-SR-01® with other robotic systems or other surgical procedures. Further analyses are still warranted.

## Conclusion

The KD-SR-01® robotic system is likely to be safe, feasible and effective for the surgical management of benign adrenal tumors and may be a good alternative clinically due to its lower cost. A multicenter comparative study with a longer follow-up is warranted to comprehensively compare the advantages and disadvantages between the KD-SR-01® robotic system and other robotic systems in urological surgeries.

## Data Availability

The original contributions presented in the study are included in the article/Supplementary Material, further inquiries can be directed to the corresponding author/s.
